# Clinicians’ attitudes towards clinical trials of cancer therapy

**DOI:** 10.1038/bjc.2011.119

**Published:** 2011-04-12

**Authors:** E Ford, V Jenkins, L Fallowfield, N Stuart, D Farewell, V Farewell

**Affiliations:** 1 Cancer Research UK Psychosocial Oncology Group, Brighton and Sussex Medical School, University of Sussex, Falmer, Brighton BN1 9QG, UK; 2 School of Medical Sciences, Brigantia Building, University of Bangor, Penrallt Road, Bangor, Wales LL57 2AS, UK; 3 Department of Primary Care and Public Health, Cardiff University School of Medicine Neuadd Meirionnydd, Heath Park, Cardiff CF14 4YS, UK; 4 MRC Biostatistics Unit, Institute of Public Health, University Forvie Site, Robinson Way, Cambridge CB2 0SR, UK

**Keywords:** clinical trials, oncologists, attitudes, recruitment, cancer therapy

## Abstract

**Background:**

Patient accrual into cancer clinical trials remains at low levels. This survey elicited attitudes and practices of cancer clinicians towards clinical trials.

**Method:**

The 43-item Clinicians Attitudes to Clinical Trials Questionnaire was completed by participants in an intervention study aimed at improving multi-disciplinary involvement in randomised trials. Responses from 13 items were summed to form a research-orientation score.

**Results:**

Eighty-seven clinicians (78%) returned questionnaires. Physicians, more often than surgeons, chose to prioritise prolonging a patient's life, recruited ⩾50% of patients into trials and attended more research-focussed conferences. Clinicians at specialist centres were more positive about trials with no-treatment arms than those at district general hospitals, more likely to believe clinician, rather than patient reluctance to participate was the greater obstacle to trial accrual, and preferred national and international to local recognition. Clinicians belonging to breast and colorectal teams were less disappointed about not enrolling patients in trials and more accepting of no-treatment arm trials. Research orientation was higher in physicians than surgeons and higher in specialist centres than district hospitals.

**Conclusions:**

This study provides greater understanding of clinicians’ attitudes to trials. Results have been used to inform training interventions for clinicians targeting the problem of low and selective accrual.

The randomised controlled trial (RCT) remains the gold-standard by which new cancer treatments are assessed and through which therapeutic progress is made. Despite the need to develop new cancer therapies, accrual of patients into such trials remains low with fewer than 5% of cancer patients participating (Tejeda *et al*, 1996; Christian and Trimble, 2003; Murthy *et al*, 2004; Somkin *et al*, 2005; Elting *et al*, 2006). Even in centres running trials suitable for a particular patient group such as women with limited, local breast cancer or other solid tumours, participation of those eligible is only 12–30% (Elting *et al*, 2006; Maslin-Prothero, 2006). As well as increasing the time needed to complete studies this low recruitment means that randomised patients may differ significantly from those who do not take part. This risks undermining the study results by making it difficult to draw generally applicable conclusions (Elting *et al*, 2006). Several US and European organisations acknowledge that slow accrual hampers the timely release of new therapies into the clinic. For example, only about 60% of US National Cancer Institute (NCI)-sponsored trials are completed and published (Nass *et al*, 2010). In addition, certain patient populations are under-represented, resulting in the recent development of several national and international programmes to address the problem of slow and selective recruitment (EDICT, 2008; Lally and Crome, 2010; Seifer *et al*, 2010).

Studies have found that the majority of clinicians favour clinical trials, viewing them as a source of high-quality patient care, and as a benefit to themselves, their institutions, and to society (Weinberg *et al*, 2004; Somkin *et al*, 2005). However, there are two main problems needing attention if trial participation is to be increased: first that clinicians do not offer trials to all eligible patients (Kaas *et al*, 2005), and second that some patients offered seemingly appropriate trials refuse participation (Siminoff *et al*, 2000). Reducing clinician gate-keeping and broadening the range of patients to whom trials are offered is challenging, but improving clinicians’ communication skills when explaining trials has the potential to resolve patients’ concerns and increase the likelihood of their participation. A review of NCI-sponsored trials noted the importance of changing physicians’ perspectives so that they will be more likely to encourage their patients to participate in clinical trials (Nass *et al*, 2010). Understanding more about clinicians’ attitudes and practices in this area is necessary and timely, if recruitment is to be improved.

Research shows that clinicians often adopt stringent, idiosyncratic criteria when selecting the patients to whom they offer trials, over and above the criteria delineated in trial protocols. In particular, some clinicians only select patients with even better health status and prognosis than demanded by the protocol (Antman *et al*, 1985; Hjorth *et al*, 1992; Rahman *et al*, 1997; Cottin *et al*, 1999; Siminoff *et al*, 2000) or choose not to offer trial participation on putative compassionate grounds (Fayter *et al*, 2007). Clinicians may hesitate to inform patients of trials, based on their own attitudes and beliefs about a patient's willingness to participate, ability to understand the trial, or adhere to the protocol (EDICT, 2008). This is despite 83% of patients being potentially willing to participate if given full trial information (Jenkins *et al*, 2010). Other clinicians are reluctant to enrol patients in trials if the treatment concerned may result in extra side effects (White *et al*, 2008). If older and sicker patients are not included in clinical trials then trial participants are likely to have better outcomes than would be seen were the new treatments offered in normal clinical practice.

Perhaps unsurprisingly, assessments of clinicians’ attitudes show that many tend to be clinically, rather than research oriented, believing that individual benefit for their patient is more important than improving future therapy (Caldwell *et al*, 2005). Some indicate that the main reason for including their patients in trials is so that they receive state of the art treatment (Joffe and Weeks, 2002), and trials focussing on quality of life outcomes tend to have better accrual rates (Hjorth *et al*, 1996).

A lack of confidence in explaining clinical trials to patients and obtaining informed consent is a barrier to some clinicians (Tournoux *et al*, 2006; Fayter *et al*, 2007). Clinicians are reticent to enrol patients who may have difficulty understanding what the trial involves (Weinberg *et al*, 2004). If the trial arms are not equally attractive or there is a no-treatment arm, this too may prove a disincentive (Maslin-Prothero, 2006). Furthermore, if the protocol is overly arduous to implement (e.g., requiring substantial time or resource commitments from the clinician or their organisation) clinicians are unlikely to recruit a large number of patients (Hjorth *et al*, 1996; Ellis *et al*, 1999; Grunfeld *et al*, 2002; Weinberg *et al*, 2004; Tournoux *et al*, 2006; Fayter *et al*, 2007). Some patients also may have a problem with understanding the concept of randomisation (Jenkins *et al*, 2002) and may struggle to accept clinical equipoise, for example, in the case of a placebo or no-treatment arm trial (Robinson *et al*, 2005). Furthermore, clinicians may explain these concepts in ways that patients may find hard to understand or even find threatening (Jenkins *et al*, 2002).

Despite these barriers, the majority of medical and clinical/radiation oncologists do report enrolling patients into clinical trials (Fallowfield *et al*, 1997). Interestingly, different specialties within oncology have different accrual rates, although which specialties accrue more is not consistent. In one survey of UK clinicians, medical oncologists generally placed more emphasis on research than did surgical or clinical oncologists, and felt greater pressure to participate in trials (Fallowfield *et al*, 1997). In Australia, medical and surgical oncologists participated more in clinical trials than radiation oncologists (Ellis *et al*, 1999). In the United States, medical and paediatric oncologists reported the lowest rates of patient enrolment in trials (Joffe and Weeks, 2002), although a more recent study showed greater recruitment among medical and radiation oncologists than among surgeons (Klabunde *et al*, 2011). A further study found no differences in attitudes towards clinical trials between surgeons and physicians, although this was not limited to oncologists (McCulloch *et al*, 2005). An understanding of how differences between specialists affect trial recruitment may help promote accrual across specialties. Some suggested reasons for differences are type of education, concept of medicine, individual or professional aims, affiliation with academic or research groups, or particular working environment (Castel *et al*, 2006), while Klabunde *et al* (2011) suggest that specialty type, involvement in teaching, affiliation with a Community Clinical Oncology Programme or with a National Cancer Institute-designated cancer centre were important.

Greater understanding of clinicians’ attitudes to clinical trials is needed to target the problem of low and selective accrual (Castel *et al*, 2006). The present survey elicited clinician attitudes towards clinical trials in their practice using a modified version of the Physician Orientation Profile (Taylor and Kelner, 1987). The aims were to assess clinician responses for differences by specialty, type of hospital, type of team, and geographic region and to estimate a research-orientation score for each clinician, again looking for differences between groups. The data used for this study are a component of a large Cancer Research UK funded prospective study examining multidisciplinary team members’ communication skills and involvement in clinical trials; primarily randomised, controlled, phase 3 trials. The main study examined different aspects of trial recruitment, including involvement of individual team members in clinical trials; assessment of the clarity of health professionals’ communication by patients recruited into clinical trials, and attitudes towards RCTs (Figure 1). The attitudes of patients to RCTs (Jenkins *et al*, 2010) and that of clinicians to RCTs were collected for each multi-disciplinary team (MDT) to provide an evidence-based argument for encouraging clinicians to consider approaching more patients about trials. The clinician data are presented here.

## Materials and methods

### Sample

Recruitment of clinicians (senior doctors) within oncology MDTs (also known as interdisciplinary tumour boards) was a joint effort between the Cancer Research UK Psychosocial Oncology Group and the Wales Cancer Trials Network. Twenty-two MDTs throughout Wales participated in the larger communication study. The study had ethical approval from the South East Wales Local Research Ethics Committee (ref: 07/WSE03/17). Teams were randomised to receive communication training or to go on a waiting list. Consultant (attending) surgeons, oncologists, chest physicians, and haematologists were asked to complete questionnaires examining their attitudes to trials and their involvement in trials before randomisation.

### Materials

The Clinicians’ Attitudes to Clinical Trials of Cancer Therapy Questionnaire is a modified version of the 30-item, binary option, Physician's Orientation Profile (Taylor and Kelner, 1987) and has been used before by this research group (Fallowfield *et al*, 1997). Items on this questionnaire are classified into five subscales assessing various aspects of clinicians’ attitudes towards their clinical and scientific work, specifically primary allegiance, professional activities, decision-making under uncertainty, perceived professional rewards, and peer group influence. The questionnaire used in this study had 43 items and is very similar to the 45-item questionnaire used by Fallowfield *et al* (1997). Professional information (specialty, MDT cancer site, etc.) was also collected for each clinician.

### Derivation of research-orientation score

Two senior researchers (LF and VJ) independently chose items from the questionnaire on the basis of their face validity for the construct ‘Research Orientation’. Each researcher independently chose 18 items from the original 43 with 100% consistency. The items were recoded where necessary to provide binary responses (0 out of 1) with 1 indicative of a research orientation. The chosen items were subjected to statistical scrutiny to assess their factorial validity and reliability. Classical principal components analysis (PCA) was undertaken based on the binary responses to these 18 questions (D’Agostino Snr, 2005) with the pragmatic aim of identifying items, which were well correlated and could be hypothesised to represent one or more underlying factors or constructs. Following PCA, a scree plot suggested a single component solution was most suitable for these items (first component eigenvalue 5.79, 32.15% of variance; second component 2.03, 11.29% of variance). Items 6, 7, 8, 12, 13, 16, 17, 27, 28, 31, 35, 36, and 40 loaded onto the first component with loadings from 0.47 to 0.83. Item 11 loaded at 0.32 and items 5, 22, 32, 43 loaded at <0.30.

All 18 items were then examined for internal consistency. Cronbach's α was found to be 0.83, and low item-total correlations were found for items 5 (0.02), 11 (0.27), 22 (0.13), 32 (0.13), and 43 (0.20), suggesting that α would increase by removing these items. Items 5, 11, 22, 32, and 43 were removed and Cronbach's α recalculated for the 13 items (α=0. 89). A second PCA on these remaining 13 items showed again that these items loaded onto one component explaining 44.4% of the variance (loadings 0.49–0.84). The responses from the remaining 13 items were summed to form a research-orientation score.

### Analysis

Question response was linked to possible explanatory variables through logistic regression. Random effects were used to account for within-team correlations, although in general these correlations were low. As the number of clinicians returning questionnaires was relatively small compared with the number of questions, and with conservatism introduced because of within-team correlations, there is little scope for multiplicity adjustments. Thus, significance levels related to individual questions should be treated with caution and seen as primarily providing an ordering of observed discriminatory ability. The analysis of the derived research-orientation score, which represented the sum of 13 questions, was based on continuation ratio ordinal regression analysis, with use of a complementary log–log link. The more flexible continuation ratio model was preferred to a binomial regression because of the variability between questions in the proportion of ‘research oriented’ answers.

## Results

Out of the 111 clinicians approached, 87 (78.4%) returned questionnaires. The sample was formed of 47 surgeons, 28 oncologists, 9 haematologists, and 3 chest physicians. These clinicians belonged to MDTs for the following cancers: breast (25; 31%), colorectal (15; 17%), gynaecology (8; 9%), haematology (10; 11.5%), lung (6; 7%), lymphoma (3; 3%), upper gastro-intestinal (10; 11.5%), and urology (10; 11.5%). Responses to each item on the questionnaire, and differences in responses by separate groupings (physician *vs* surgeon; specialist hospital (large teaching or city hospital) *vs* district general hospital (DGH) (community hospital); breast and colorectal teams *vs* others) are shown in Table 1.

### Differences by specialty

Table 2 shows the questionnaire items that best distinguished between physicians (oncologists, haematologists, and chest physicians) and surgeons. Physicians more frequently endorsed prolonging a patient's life over improving quality of life. They were also more likely to report recruiting 50% or more of their patients into clinical trials and attending conferences focussed on research rather than clinical issues. These differences suggest physicians may be more research focussed than their surgical counterparts.

### Differences by centre

Table 3 shows the questionnaire items best differentiating clinicians who worked in a specialist cancer centre from those who worked in a DGH. Those working at a specialist centre were less reluctant to randomise patients into trials with no-treatment arms, more likely to believe that clinician reluctance (as opposed to patient reluctance) to participate was a major obstacle to trial accrual, and were more likely to wish to be well known among national or international, rather than local, colleagues. These differences suggest that those at specialist centres may be more research oriented than those at DGHs.

### Differences by geographical region

There were some regional differences in answers to questionnaire items, but after exploration of confounders, it was found that the effect of region (SE *vs* N and SW) disappeared when specialist centre was added to the model.

### Differences by type of MDT

Multi-disciplinary teams were grouped into breast and colorectal teams *vs* the other teams (haematology, gynaecology, lung, lymphoma, upper gastro-intestinal, and urology). This grouping was chosen because breast and colorectal cancer are areas where there are typically many clinical trials and where recruitment has been common (UK Clinical Research Network, 2010). We hypothesised that MDTs treating such patients may be more favourable to clinical research. Table 4 shows the questionnaire items which best differentiate clinicians by their MDT specialty. These results suggest that breast and colorectal teams may be less disappointed about not enrolling patients in a trial and more accepting of trials with no-treatment arms. Interestingly, clinicians belonging to other types of teams reported stronger publications records and were more desirous of international rather than local recognition.

### Research orientation

Binary responses to questions 6, 7, 8, 12, 13, 16, 17, 27, 28, 31, 35, 36, and 40 (highlighted in bold in Table 1) were summed to form the research-orientation scale. Full data were available from 74 participants and scores on the scale ranged from 0 to 12. The numbers of observations with these 13 values were 18, 23, 7, 8, 4, 4, 1, 1, 1, 2, 3, 1, and 1, respectively, showing a skew towards the non-research orientation end of the scale. Ordinal regression analysis demonstrated that the probability of a high research orientation was greater in physicians *vs* surgeons (*P*<0.001) and in respondents from a specialist centre (*P*<0.001) but little demonstrable effect was associated with MDT type (breast/colorectal *vs* rest: *P*=0.06). Both the physician/surgeon and specialist/DGH centre classifications retained their significance in a multivariate analysis but MDT type demonstrated no relationship with research orientation (Table 5).

## Discussion

Recruitment of patients into clinical trials is essential if progress is to be made in improving cancer care. Despite this, recruitment is often poor with studies slow to complete and with only a small proportion of cancer patients being enrolled in studies. The process of recruitment involves discussion between the patient and their doctor but is preceded by identification of suitable patients fulfilling eligibility criteria and then the doctor's decision to offer the patient an appropriate trial. The attitude of doctors is therefore a critical part of the enrolment process.

This study evaluated doctors’ attitudes to clinical research and to clinical trial recruitment. Using the Clinicians’ Attitudes to Clinical Trials of Cancer Therapy Questionnaire the attitudes of 87 clinicians with a range of specialties, tumour site interests, geographical location, and affiliations were assessed. The questionnaire also allowed clinicians to be allocated a research orientation score.

The results indicate that research orientation was greater in physicians than surgeons, with physicians more likely to enter patients into trials and more likely to attend research-focussed conferences. The lesser research orientation found in surgeons may reflect the paucity of surgical trials available and the difficulties encountered recruiting patients into a surgical trial. One study of US breast cancer surgeons showed that 26% did not discuss trials with any breast cancer patients, and a further 39% discussed trials with fewer than 10%. Surgeons identified inadequate infrastructure and lack of time as significant deterrents in trial participation (Schroen and Brenin, 2008). Klabunde *et al* (2011) also show significantly lower clinical trial recruitment among surgeons compared with oncologists although surgical oncologists were more likely to participate in clinical trials than general surgeons. Furthermore, surgeons showed the same pattern as physicians with increased trial participation being associated with academic affiliation, teaching of medical students, having a more specialist clinical practice, and more frequent attendance at multidisciplinary meetings.

It may be that although surgeons are a crucial part of a cancer team, they feel they have a remote role in recruiting patients to medical/oncological trials. The increasing number of neo-adjuvant and peri-operative trials makes this untenable. Furthermore, it can be argued that the surgeon has a pivotal role in influencing patients’ expectations about the cancer treatments on offer following surgery. The way the surgeon introduces the idea of trials in general can facilitate any discussions, which follow with the oncologist. If trials are treated as a ‘team business’, all team members should be made aware of what trials are available for patients in that tumour site and have the skills and knowledge necessary to discuss these trials with patients.

The finding that clinicians at specialist centres are more research oriented than those in DGHs is not surprising. Specialist centres have more staff and resources available for trials and are more likely to be set up to facilitate the running of trials. For example, in a DGH there may be only one histopathologist dealing with several cancer sites and who does not have any protected time to deal with ‘trial’ tissue blocks in the tight timelines that some trials have. Specialist centres may also be attached to research institutions and therefore their staff is more likely to have a teaching or research focus. Research-oriented clinicians may self-select into posts within specialist centres or teaching hospitals. It is interesting that clinicians working in specialist centres seem more aware that clinician reluctance could be a barrier to the successful completion of a clinical trial. This may result in clinicians in specialist centres being more aware of their communication training needs and more likely to address their own personal barriers to trial recruitment.

Furthermore, ability to find trial information, and the familiarity of investigators with trials, may differ in different types of hospital. According to one survey, the most common reason cited for physician non-participation in trials was a lack of knowledge about available trials (Taylor, 2004). A review of US publicly funded trials suggested that ‘encouraging the development of a user-friendly, transparent, up-to-date, and easily accessible centralised registry could improve both physician and patient awareness of the available trials. In combination with electronic tools, such as, clinical decision support software, a centralised registry could cue physicians to important, applicable clinical trials at the point of care’ (Nass *et al*, 2010, p. 204). A centralised system of trial information available to all clinicians, whatever type of institution they worked in, would potentially overcome some barriers to recruitment in DGHs.

Lack of clinical trial support has been frequently cited as a barrier to clinical trial involvement (Somkin *et al*, 2005; Schroen and Brenin, 2008) but this issue does not explain the findings in this study as all the MDTs involved in the study were supported by the Wales Cancer Trials Network whose infrastructure (research nurse time, data management support, etc.) was available to all team members. Other papers refer to lack of reimbursement for clinical trial work and other financial constraints as a barrier to recruitment (Klabunde *et al*, 2011). Again this was not relevant to this study, which took place in a health service (NHS) where all staff are salaried.

Our hypothesis that clinicians working in breast and colorectal cancer teams would be more research oriented was not borne out by the data. Although these clinicians were more accepting of no-treatment arms and more sanguine about patients declining trial entry, they were less likely to seek national or international recognition and had fewer publications compared with clinicians treating other cancer sites. It appears then that specialty by cancer site is not a marker of research orientation. As new therapies are required across all types of cancer, it may be that clinicians specialising in all tumour sites have a broadly equal interest in research, because of the constantly emerging treatments in their field.

The research-orientation scale showed a strong skew towards the non-research orientation end of the scale, with the majority of participants scoring 0 or 1 out of a possible 13. However, despite the skew, in ordinal analyses scores on this scale were significantly different between surgeons and physicians, and between those working in specialist centres and in DGHs, confirming our findings from the single question analyses. This scale can therefore be used in future studies to investigate clinician orientation towards research in general and trials in particular, and could potentially be used to evaluate training materials designed to increase research interest and participation among clinicians.

There are several limitations to this study. The clinicians assessed were all members of recognised cancer MDTs and were involved in regular discussion of patient management. In Wales, all clinical trial recruitment is recorded centrally and MDTs with no recognised trial recruitment were excluded as this was an explicit entry criterion for the associated trial. It is therefore possible that the attitudes expressed are more favourable towards research than would be seen in the general clinical population, as MDTs with no trial experience were excluded.

Second, the questions on attitudes in this study asked primarily about attitudes to RCTs and not to earlier, phase 1 and 2 trials. Clinicians may have different attitudes to early phase trials, which have different aims and require specialist infrastructure. Third, only a small range of clinician characteristics, such as specialty, were available. Clinician attitudes may also vary by other clinician characteristics, such as number of patients seen per month, clinician sex, training status, previous experience as principal investigator, time since graduation, or academic or industry affiliations (Joffe and Weeks, 2002). In addition, the clinician sample in this study was culturally homogenous and therefore cultural or ethnic differences in attitudes could not be explored. Findings may be different in a more multicultural sample. It is also possible that clinician attitudes may depend on the sociodemographic characteristics of their patient population. Further research in this area would be interesting and may be useful in increasing understanding about current recruitment practices.

In conclusion, this study gives us greater understanding of clinicians’ attitudes to clinical trials, in order to target the problem of low and selective accrual. Despite an increase in overall recruitment to trials over the past 5 years, patients’ understanding of them and health professionals’ explanations about them are still problematic. There is a crucial need for all cancer team members to be fully supportive and aware of the trials that are available in their centres, and for all to receive and deliver consistent information to patients and their relatives. The results of this study are part of a larger project, in which members from participating MDTs have taken part in training. The training has addressed problems such as team dynamics acting as a barrier to trial recruitment, inconsistent team communication, and the difficulty of talking about trials where patients are unenthusiastic about joining for different reasons. Previous research has shown that communication skills training can also alter the attitudes and beliefs of clinicians (Jenkins and Fallowfield, 2002). The overall aims of this work are to increase the number of patients offered a trial by clinicians, and to increase the quality of communication about trials when they are discussed.

## Figures and Tables

**Figure 1:**
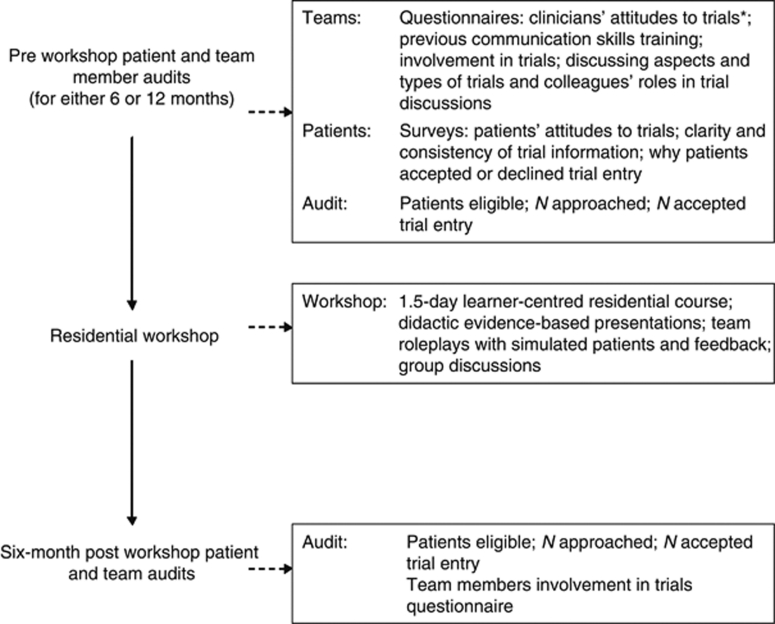
Overall ‘Teams Talking About Trials’ project. ^*^Data presented in this paper.

**Table 1:** Differences in response in physicians *vs* surgeons, specialist hospital *vs* district general and breast and colorectal teams *vs* other teams

	**Overall Sample (%)**	**Physician (%) (*n*=40)**	**Surgeon (%) (*n*=47)**	**DGH (%) (*n*=33)**	**Specialist (%) (*n*=54)**	**Breast/colorectal (%) (*n*=40)**	**Other cancer type (%) (*n*=47)**
*Q1. Ideally, clinicians are able to increase survival and improve the quality of patients’ lives. In cases where only one can be achieved at the cost of the other, I feel more satisfied when I can:*							
(a) improve patients’ quality of life	71 (83.5%)	29 (74.3)	42 (91.3)	27 (84.4)	44 (83.0)	32 (82.1)	39 (84.8)
(b) prolong patients’ lives	14 (16.5%)	10 (25.6)	4 (8.7)	5 (15.6)	9 (17.0)	7 (17.9)	7 (15.2)
							
*Q2. If a patient refuses to participate in a randomised clinical trial that I suggest, I would:*							
(a) treat the patient off the study	87 (100%)	—	—	—	—	—	—
(b) refer the patient to another clinician	0 (0%)	—	—	—	—	—	—
							
*Q3. In general, when I initiate a treatment for cancer, I am:*							
(a) optimistic that the treatment will work	80 (93.0%)	38 (97.4)	42 (89.4)	31 (93.9)	49 (92.5)	37 (92.5)	43 (93.4)
(b) pessimistic that the treatment will work	6 (7.0%)	1 (2.6)	5 (10.6)	2 (6.1)	4 (7.5)	3 (7.5)	3 (6.5)
							
*Q4. In my hospital the pressure to participate in a randomised clinical trial is relatively:*							
(a) low	58 (68.2%)	21(53.8)	37 (80.4)	26 (78.8)	32 (61.5)	29 (74.4)	29 (63.0)
(b) high	27 (31.8%)	18 (46.2)	9 (19.6)	7 (21.2)	20 (38.5)	10 (25.6)	17 (37.0)
							
*Q5. I enter the following amount of my potentially eligible patients into randomised clinical trials:*							
(a) under 50%	66 (75.9%)	25 (62.5)	41 (87.2)	27 (81.8)	39 (72.2)	31 (77.5)	35 (74.5)
(b) 50% or more	21 (24.1%)	15 (37.5)	6 (12.8)	6 (18.2)	15 (27.8)	9 (22.5)	12 (25.5)
							
***Q6. My primary commitment is to:***							
**(a) future generations of patients (society)**	**9** **(10.7%)**	**4** **(10.3)**	**5** **(11.1)**	**3** **(9.1)**	**6** **(11.8)**	**7** **(18.4)**	**2** **(4.3)**
**(b) present patients (individuals)**	**75** **(89.3%)**	**35** **(89.7)**	**40** **(88.9)**	**30** **(90.9)**	**45** **(88.2)**	**31** **(81.6)**	**44** **(95.6)**
							
***Q7. When faced with a controversial treatment decision, I feel most comfortable when:***							
**(a) I make the decisions**	**11** **(12.8%)**	**4** **(10.3)**	**7** **(14.9)**	**4** **(12.1)**	**7** **(13.2)**	**6** **(15.0)**	**5** **(10.9)**
**(b) the decisions are made by the trial protocol**	**8** **(9.3%)**	**4** **(10.3)**	**4** **(8.5)**	**2** **(6.1)**	**6** **(11.3)**	**3** **(7.5)**	**5** **(10.9)**
**(c) the decision is made by the Multi-Disciplinary Meeting**	**67** **(77.9%)**	**31** **(79.5)**	**36** **(76.6)**	**27** **(81.8)**	**40** **(75.5)**	**31** **(77.5)**	**36** **(78.3)**
							
***Q8. Currently, I am the principal investigator on one or more research grants:***							
**(a) no**	**55** **(63.2%)**	**22** **(55.0)**	**33** **(70.2)**	**26** **(78.8)**	**29** **(53.7)**	**27** **(67.5)**	**28** **(59.6)**
**(b) yes**	**32** **(36.7%)**	**18** **(45.0)**	**14** **(29.8)**	**7** **(21.2)**	**25** **(46.3)**	**13** **(32.5)**	**19** **(40.4)**
							
*Q9. In my hospital, doctors are given more reward for:*							
(a) clinical skills with patients	63 (79.7%)	28 (75.7)	35 (83.3)	29 (87.9)	34 (73.9)	29 (82.9)	34 (77.3)
(b) contributing to scientific knowledge	16 (20.3%)	9 (24.3)	7 (16.7)	4 (12.1)	12 (26.1)	6 (17.1)	10 (22.7)
							
*Q10. When a patient on a protocol relapses or progresses and the protocol dictates additional treatment that the patient does not want, I:*							
(a) encourage the patient to stay on the trial	28 (33.7%)	11 (28.2)	17 (38.6)	11 (33.3)	17 (34.0)	13 (35.1)	15 (32.6)
(b) remove the patient from the trial	55 (66.3%)	28 (71.8)	27 (61.4)	22 (66.7)	33 (66.0)	24 (64.9)	31 (67.4)
							
*Q11. In general, patients are referred to me because of my:*							
(a) research activities	3 (3.5%)	2 (5.1)	1 (2.1)	1 (3.0)	2 (3.8)	2 (5.0)	1 (2.2)
(b) clinical reputation	83 (96.5%)	37 (94.9)	46 (97.9)	32 (97.0)	51 (96.2)	38 (95.0)	45 (97.8)
							
***Q12. I participate more actively in professional organisations that are based on:***							
**(a) my clinical speciality**	**74** **(87.1%)**	**29** **(76.3)**	**45** **(95.7)**	**32** **(97.0)**	**42** **(80.8)**	**35** **(89.7)**	**39** **(84.8)**
**(b) my research activities**	**11** **(12.9%)**	**9** **(23.7)**	**2** **(4.3)**	**1** **(3.0)**	**10** **(19.2)**	**4** **(10.3)**	**7** **(15.2)**
							
***Q13. The time I devote to publications, lectures and research commitments, compared with clinical work, is relatively:***							
**(a) low**	**74** **(86.0%)**	**31** **(79.5)**	**43** **(91.5)**	**32** **(97.0)**	**42** **(79.2)**	**34** **(85.0)**	**40** **(87.0)**
**(b) high**	**12** **(14.0%)**	**8** **(20.5)**	**4** **(8.5)**	**1** **(3.0)**	**11** **(20.8)**	**6** **(15.0)**	**6** **(13.0)**
							
*Q14. The need for detailed monitoring of individual clinicians’ activities deters me from participating in randomised clinical trials:*							
(a) no	72 (82.8%)	37 (92.5)	35 (74.5)	24 (72.7)	48 (88.9)	32 (80.0)	40 (85.1)
(b) yes	15 (17.2%)	3 (7.5)	12 (25.5)	9 (27.3)	6 (11.1)	8 (20.0)	7 (14.9)
							
*Q15. When a potentially eligible patient chooses not to enrol on a trial that I have suggested, I:*							
(a) often feel disappointed	26 (30.2%)	11 (27.5)	15 (32.6)	10 (30.3)	16 (30.2)	6 (15.4)	20 (42.6)
(b) seldom feel disappointed	60 (69.8%)	29 (72.5)	31 (67.4)	23 (69.7)	37 (69.8)	33 (84.6)	27 (57.4)
							
***Q16. I devote a lot of time to educating other clinicians about randomised clinical trials:***							
**(a) no**	**60** **(69.0%)**	**21** **(52.5)**	**39** **(83.0)**	**27** **(81.8)**	**33** **(61.1)**	**27** **(67.5)**	**33** **(70.2)**
**(b) yes**	**27** **(31.0%)**	**19** **(47.5)**	**8** **(17.0)**	**6** **(18.2)**	**21** **(38.9)**	**13** **(32.5)**	**14** **(29.8)**
							
***Q17. Frequent publications are important to my career advancement:***							
**(a) agree**	**45** **(52.3%)**	**21** **(52.5)**	**24** **(52.2)**	**14** **(42.4)**	**31** **(58.5)**	**22** **(56.4)**	**23** **(48.9)**
**(b) disagree**	**41** **(47.7%)**	**19** **(47.5)**	**22** **(47.8)**	**19** **(57.6)**	**22** **(41.5)**	**17** **(43.6)**	**24** **(51.1)**
							
*Q18. When a protocol includes a treatment that is more aggressive than I would usually give to similar non-trial patients:*							
(a) I am often reluctant to participate	27 (31.4%)	14 (35.0)	13 (28.3)	8 (24.2)	19 (35.8)	11 (28.2)	16 (34.0)
(b) it makes no difference	59 (68.6%)	26 (65.0)	33 (71.7)	25 (75.8)	34 (64.2)	28 (71.8)	31 (66.0)
							
*Q19. I am reluctant to participate in a trial that may randomise the patient to a ‘no treatment’ group:*							
(a) agree	18 (20.7%)	5 (12.5)	13 (27.7)	11 (33.3)	7 (13.0)	14 (35.0)	4 (8.5)
(b) disagree	69 (79.3%)	35 (87.5)	34 (72.3)	22 (66.7)	47 (87.0)	26 (65.0)	43 (91.5)
							
*Q20. After being randomised, if a patient refuses the treatment to which he or she has been assigned:*							
(a) I accept the patient's decision	71 (81.6%)	28 (70.0)	43 (91.5)	28 (84.8)	43 (79.6)	35 (87.5)	36 (76.6)
(b) I make every effort to keep the patient on the trial	16 (18.4%)	12 (30.0)	4 (8.5)	5 (15.1)	11 (20.4)	5 (12.5)	11 (23.4)
							
*Q21. Overall I feel the quality of patient care:*							
(a) increases when patient is in a clinical trial	83 (98.8%)	40 (100)	43 (97.7)	30 (96.8)	53 (100)	38 (97.4)	45 (100)
(b) decreases when patient is in a clinical trial	1 (1.2%)	0 (0)	1 (2.3)	1 (3.2)	0 (0)	1 (2.6)	0 (0)
							
*Q22. When published data and my clinical experience conflict, I am more likely to rely on:*							
(a) my clinical experience	49 (59.0%)	20 (52.6)	29 (64.4)	22 (71.0)	27 (51.9)	24 (61.5)	25 (56.8)
(b) published data	34 (41.0%)	18 (47.4)	16 (35.6)	9 (29.0)	25 (48.1)	15 (38.5)	19 (43.2)
							
*Q23. The more frequent obstacle to the successful completion of a clinical trial is:*							
(a) clinicians’ reluctance to participate	57 (67.1%)	27 (67.5)	30 (66.7)	17 (53.1)	40 (75.4)	26 (68.4)	31 (66.0)
(b) patients’ reluctance to participate	28 (32.9%)	13 (32.5)	15 (33.3)	15 (46.9)	13 (24.5)	12 (31.6)	16 (34.0)
							
*Q24. If written informed consent were not required, I would approach more patients to enter clinical trials:*							
(a) true	16 (18.4%)	5 (12.5)	11 (23.4)	5 (15.1)	11 (20.4)	10 (25.0)	6 (12.8)
(b) false	71 (81.6%)	35 (87.5)	36 (76.6)	28 (84.8)	43 (79.6)	30 (75.0)	41 (87.2)
							
*Q25. The opinions of the referring clinician regarding randomised clinical trials affects my decision to approach an eligible patient:*							
(a) true	14 (16.3%)	5 (12.5)	9 (19.6)	5 (15.1)	9 (17.0)	8 (20.5)	6 (12.8)
(b) false	72 (83.7%)	35 (87.5)	37 (80.4)	28 (84.8)	44 (83.0)	31 (79.5)	41 (87.2)
							
*Q26. The thought of having to spell out all the details of a trial to eligible patients discourages me from approaching them to participate:*							
(a) true	20 (23.0%)	7 (17.5)	13 (27.7)	9 (27.3)	11 (20.4)	13 (32.5)	7 (14.9)
(b) false	67 (77.0%)	33 (82.5)	34 (72.3)	24 (72.7)	43 (79.6)	27 (67.5)	40 (85.1)
							
***Q27. It is more important for me to be well-known among:***							
**(a) local colleagues**	**56** **(70.0%)**	**24** **(63.2)**	**32** **(76.2)**	**28** **(90.3)**	**28** **(57.1)**	**28** **(82.4)**	**28** **(60.9)**
**(b) national/international colleagues**	**24** **(30.0%)**	**14** **(36.8)**	**10** **(23.8)**	**3** **(9.7)**	**21** **(42.9)**	**6** **(17.6)**	**18** **(39.1)**
							
***Q28. I spend the following amount of my time in research-related activities:***							
**(a) less than one-third**	**75** **(86.2%)**	**31** **(77.5)**	**44** **(93.6)**	**33** **(100)**	**42** **(77.8)**	**35** **(87.5)**	**40** **(85.1)**
**(b) one-third or more**	**12** **(14.2%)**	**9** **(22.5)**	**3** **(6.4)**	**0** **(0)**	**12** **(22.2)**	**5** **(12.5)**	**7** **(14.9)**
							
*Q29. A major reason for my participation in randomised clinical trials is that it benefits my institution:*							
(a) agree	43 (49.4%)	20 (50)	23 (48.9)	14 (42.4)	29 (53.7)	21 (52.5)	22 (46.8)
(b) disagree	44 (50.6%)	20 (50)	24 (51.1)	19 (57.6)	25 (46.3)	19 (47.5)	25 (53.2)
							
*Q30. Overall, participation in a randomised clinical trial is:*							
(a) an asset to my reputation	84 (100%)	—	—	—	—	—	—
(b) a liability to my reputation	0 (0%)	—	—	—	—	—	—
							
***Q31. If I could have only one measure, I would assess how successful I was as a physician by:***							
**(a) my research contributions**	**12** **(14.0%)**	**8** **(20.5)**	**4** **(8.5)**	**1** **(3.0)**	**11** **(20.8)**	**5** **(12.5)**	**7** **(15.2)**
**(b) how I helped individual patients**	**74** **(86.0%)**	**31** **(79.5)**	**43** **(91.5)**	**32** **(97.0)**	**42** **(79.2)**	**35** **(87.5)**	**39** **(84.8)**
							
*Q32. When I am personally uncertain as to which treatment is best, I am likely to:*							
(a) enter the patient in a randomised clinical trial if one exists	74 (86.0%)	38 (95.0)	36 (78.3)	27 (84.4)	47 (87.0)	33 (82.5)	41 (89.1)
(b) personally select a treatment	12 (14.0%)	2 (5.0)	10 (21.7)	5 (15.6)	7 (13.0)	7 (17.5)	5 (10.9)
							
*Q33. When I obtain informed consent:*							
(a) I allow patient reaction to influence the content of the information given	50 (57.5%)	22 (55.0)	28 (59.6)	22 (66.7)	28 (51.9)	25 (62.5)	25 (53.2)
(b) I do not vary the content of the information given	37 (42.5%)	18 (45.0)	19 (40.4)	11 (33.3)	26 (48.1)	15 (37.5)	22 (46.8)
							
*Q34. If research activities were to enhance my income, I would enter more patients in randomised clinical trials:*							
(a) agree	28 (32.5%)	12 (30.0)	16 (34.8)	12 (36.4)	16 (30.2)	14 (35.9)	14 (29.8)
(b) disagree	58 (67.5%)	28 (70.0)	30 (65.2)	21 (63.6)	37 (69.8)	25 (64.1)	33 (70.2)
							
***Q35. I am more likely to attend a conference that focuses on:***							
**(a) clinical issues**	**61** **(76.3%)**	**23** **(62.2)**	**38** **(88.4)**	**28** **(90.3)**	**33** **(67.3)**	**27** **(77.1)**	**34** **(75.6)**
**(b) research issues**	**19** **(23.7%)**	**14** **(37.8)**	**5** **(11.6)**	**3** **(9.7)**	**16** **(32.6)**	**8** **(22.9)**	**11** **(24.4)**
							
***Q36. In the past 3 years, I have authored/co-authored:***							
**(a) 0 publications**	**12** **(13.7%)**	**9** **(22.5)**	**3** **(6.4)**	**8** **(24.2)**	**4** **(7.4)**	**7** **(17.5)**	**5** **(10.6)**
**(b) 1–5 publications**	**51** **(58.6%)**	**19** **(47.5)**	**32** **(68.1)**	**21** **(63.6)**	**30** **(55.6)**	**27** **(67.5)**	**24** **(51.1)**
**(c) 6–9 publications**	**14** **(16.1%)**	**7** **(17.5)**	**7** **(14.9)**	**2** **(6.1)**	**12** **(22.2)**	**4** **(10.0)**	**10** **(21.3)**
**(d) 10 or more publications**	**10** **(11.4%)**	**5** **(12.5)**	**5** **(10.6)**	**2** **(6.1)**	**8** **(14.8)**	**2** **(5.0)**	**8** **(17.0)**
							
*Q37. When informing patients about their prognosis, I find statistics:*							
(a) helpful	78 (90.7%)	36 (92.3)	42 (89.4)	28 (87.5)	50 (92.6)	36 (90.0)	42 (91.3)
(b) not helpful	8 (9.3%)	3 (7.7)	5 (10.6)	4 (12.5)	4 (7.4)	4 (10.0)	4 (8.7)
							
*Q38. When making critical and controversial decisions I usually:*							
(a) seek major input from my patients	83 (96.5%)	39 (97.5)	44 (95.7)	33 (100)	50 (94.3)	37 (94.9)	46 (97.9)
(b) do not seek major input from my patients	3 (3.5%)	1 (2.5)	2 (4.3)	0 (0)	3 (5.7)	2 (5.1)	1 (2.1)
							
*Q39. I think the patient's right to select treatment options is always more important than the advancement of scientific knowledge:*							
(a) true	73 (83.9%)	32 (80.0)	41 (87.2)	25 (75.8)	48 (88.9)	33 (82.5)	40 (85.1)
(b) false	14 (16.1%)	8 (20.0)	6 (12.8)	8 (24.2)	6 (11.1)	7 (17.5)	7 (14.9)
							
***Q40. If I had to choose, I would say my primary task is:***							
**(a) caring for individual patients**	**82** **(95.3%)**	**36** **(92.3)**	**46** **(97.9)**	**33** **(100)**	**49** **(92.5)**	**38** **(95.0)**	**44** **(95.7)**
**(b) contributing to scientific knowledge**	**4** **(4.6%)**	**3** **(7.7)**	**1** **(2.1)**	**0** **(0)**	**4** **(7.5)**	**2** **(5.0)**	**2** **(4.3)**
							
*Q41. I would rather be somewhat:*							
(a) too involved with my patients	75 (87.2%)	37 (92.5)	38 (82.6)	26 (81.25)	49 (90.7)	34 (87.2)	41 (87.2)
(b) too detached from my patients	11 (12.8%)	3 (7.5)	8 (17.4)	6 (18.75)	5 (9.3)	5 (12.8)	6 (12.8)
							
*Q42. When I participate in a randomised clinical trial, it is more likely that I:*							
(a) increase my patient population	72 (91.1%)	36 (94.7)	36 (87.8)	26 (86.7)	46 (93.9)	32 (91.4)	40 (90.9)
(b) lose patients I might otherwise keep	7 (8.9%)	2 (5.3)	5 (12.2)	4 (13.3)	3 (6.1)	3 (8.6)	4 (9.1)
							
*Q43. When there is controversy in the literature as to which treatment is best:*							
(a) I enter the patient in a clinical trial if one exists	79 (90.8%)	38 (95.0)	41 (87.2)	28 (84.8)	51 (94.4)	34 (85.0)	45 (95.7)
(b) I personally select a treatment for the patient	8 (9.2%)	2 (5.0)	6 (12.8)	5 (15.2)	3 (5.6)	6 (15.0)	2 (4.3)

Abbreviation: DGH=district general hospital.

Items highlighted in bold are used in the research-orientation score.

**Table 2:** Items distinguishing between physicians and surgeons

**Questionnaire Item**	**Physicians**	**Surgeons**	**Beta (sig.)**
*Q1. Ideally, clinicians are able to increase survival and improve the quality of patients’ lives. In cases where only one can be achieved at the cost of the other, I feel more satisfied when I can:*			
(a) improve patients’ quality of life	29 (74%)	42 (91%)	1.29 (0.04)
(b) prolong patients’ lives	10 (26%)	4 (9%)	
			
*Q5. I enter the following amount of my potentially eligible patients into randomised clinical trials:*			
(a) under 50%	25 (62.5%)	41 (87%)	1.44 (0.02)
(b) 50% or more	15 (37.5%)	6 (13%)	
			
*Q35. I am likely to attend a conference that focuses on:*			
(a) clinical issues	23 (62%)	38 (88%)	1.63 (0.006)
(b) research issues	14 (38%)	5 (12%)	

**Table 3:** Items distinguishing between clinicians in specialist centres and those in district general hospitals

**Questionnaire Item**	**DGH**	**Specialist centre**	**Beta (sig.)**
*Q19. I am reluctant to participate in a trial that may randomise the patient to a ‘no treatment’ group:*			
(a) agree	11 (33%)	7 (13%)	1.21 (0.03)
(b) disagree	22 (67%)	47 (87%)	
			
*Q23. The more frequent obstacle to the successful completion of a clinical trial is:*			
(a) clinicians’ reluctance to participate	17 (53%)	40 (76%)	−1.00 (0.04)
(b) patients’ reluctance to participate	15 (47%)	13 (24%)	
			
*Q27. It is more important for me to be well known among:*			
(a) local colleagues	28 (90%)	28 (57%)	1.95 (<0.004)
(b) national/international colleagues	3 (10%)	21 (43%)	

Abbreviation: DGH=district general hospital.

**Table 4:** Items distinguishing between MDTs

**Questionnaire item**	**Breast and colorectal**	**Others**	**Beta (sig.)**
*Q15. When a potentially eligible patient chooses not to enrol on a trial that I have suggested I:*			
(a) often feel disappointed	6 (15%)	20 (43%)	1.40 (0.0008)
(b) seldom feel disappointed	33 (85%)	27 (57%)	
			
*Q19. I am reluctant to participate in a trial that may randomise a patient to a ‘no treatment’ group.*			
(a) agree	14 (35%)	4 (09%)	−1.76 (0.005)
(b) disagree	26 (65%)	43 (91%)	
			
*Q36. In the past 3 years, I have authored/co-authored:*			
(a) 0	7 (17%)	5 (11%)	(a)(b) *vs* (c)(d)−1.38 (0.03)
(b) 1–5	27 (68%)	24 (51%)	
(c) 6–9	4 (10%)	10 (21%)	
(d) 10+	2 (5%)	8 (17%)	
			
*Q27. It is more important for me to be well known among:*			
(a) local colleagues	28 (82%)	28 (61%)	−1.10 (0.04)
(b) national/international colleagues	6 (18%)	18 (39%)	

Abbreviation: MDT=multi-disciplinary team.

**Table 5:** Multivariate ordinal regression analysis of clinical/research-orientation scale

**Variable**	**Coefficient**	**Standard error**	**Significance level**
Physician/surgeon	0.69	0.11	<0.001
Specialist/DGH	0.95	0.13	<0.001
Breast/colorectal *vs* other	0.03	0.11	0.78

Abbreviation: DGH= district general hospital.
